# The community psychosocial burden during the COVID-19 pandemic in Indonesia

**DOI:** 10.1016/j.heliyon.2020.e05136

**Published:** 2020-09-30

**Authors:** Hario Megatsari, Agung Dwi Laksono, Mursyidul Ibad, Yeni Tri Herwanto, Kinanty Putri Sarweni, Rachmad Ardiansyah Pua Geno, Estiningtyas Nugraheni

**Affiliations:** a Faculty of Public Health, Universitas Airlangga, Surabaya, Indonesia; b National Institute of Health Research and Development, The Indonesian Ministry of Health, Jakarta, Indonesia; c Faculty of Health, Nadlatul Ulama University, Surabaya, Indonesia; d The Indonesian Public Health Union, Makassar, Indonesia

**Keywords:** Psychology, Public health, Mental health, Psychosocial burden, Health behaviour, COVID-19

## Abstract

**Background:**

Restricting community mobility during COVID-19 can potentially trigger anxiety, depression and stress in the community. The study aims to analyze variables associated with the community psychosocial burden (anxiety level) during the co-19 pandemic in Indonesia.

**Methods:**

This study collected data (n = 8,031) online. Psychosocial burden was measured based on the anxiety level which include 5 aspects, such as economic, religious, educational, employment, and social issues. Each question used a Likert scale. Six independent were examined, such as age, gender, religion, marital, education, and employement. In the final stage, a multivariate test was performed using a multinomial logistic regression.

**Results:**

Someone older experienced less high anxiety. The age group of 20–29 years was 4,330 times likely to experience higher anxiety than the age group of ≥50 years. While, those in the age group of 40–49 years weer 2,322 times more likely to have higher anxiety than those in the age group of ≥50 years. Male respondents had lower possibility of medium to high anxiety than females. Respondents with secondary and lower education had 3,117 times possibilities to experience higher anxiety than those with high education level.

**Conclusion:**

Four variables affected the psychosocial burden i.e, anxiety level of community in Indonesia. These involved age, gender, education, and employement.

## 1 Introduction

Indonesia so far has not shown a decline in COVID-19 cases. Data accessed on 15 June 2020 from the official website of the Indonesian government, www.covid19.go.id, illustrates that the distribution of COVID-19 cases has been fairly distributed in all provinces and districts/cities. Some regions have indicated a new case. In other words, it has not indicated that this outbreak will end soon.

The government has taken several strategies to break the chain of COVID-19 transmission. For example, it issued the Regulation of Indonesian Minsitry of Health No. 9 of 2020 concerning Large-Scale Social Limitation Guidelines in accelerating COVID-19 management. It regulates some restrictions for the community, such as in school hours, operation of public transportation, work from home, and so forth. This regulation limits the mobility of community (Task Force for the Acceleration of Handling COVID-19, 2020a; the Minister of Health of the Republic of Indonesia, 2020).

The regulation and restrictions have affected all aspects, such as religious, economy, education and social psychology. Mass media reported that some people still have to work outside even though the local government has reinforced the regulation (Laksono et al., 2020). A study found longer large scale social restrictions might result in more violations in the community (Ibad et al., 2020). Another phenomenon is that some religious groups still gather to perform religious rituals in which some new cases potentially can occur.

The limitation of community mobility during COVID-19 can potentially trigger anxiety, depression and stress in the society (Bohlken et al., 2020). Several factors that affect anxiety include environmental, emotional, and physical factors. Anxiety can arise as people are urged to stay at home. It, therefore, results in a domino effect due to the suppressed situation. For example, people cannot do daily activities like normally, interact with their social group, perform religious activities outside, and so on (Mukhtar and Rana, 2020).

Several recent studies in some countries have reported anxiety which resulted in psychosocial anomalies during the COVID-19 pandemic. This situation can lead to a negative situation, such as decreasing immunity. During the COVID-19 pandemic, people should maintain their immunity (Lima et al., 2020; Mukhtar and Rana, 2020; Rajkumar 2020). One study even reported high anxiety could lead to suicide incidents during COVID-19 pandemic (Sher, 2020).

Anxiety is also caused by the abundance of hoax information on social media, and eventually it can worsen the community pshycological condition (Athbi and Hassan, 2019; Costa, 2019; Evans, 2020). Such worse situation might be originated from the opinion of celebrities and influencers about the theory of conspiracy on the COVID-19 pandemic (Task Force for the Acceleration of Handling COVID-19 2020b). For example, the president of the USA, Donald Trump, believed that the pandemic was brought by the theory of conspiracy as a form of resistance to the power of government (Dyer, 2020).

Public anxiety can lead to new public health problems. The WHO defines health as a good condition seen from physical, social and mental dimensions (World Health Organization, 1946). Anxiety is considered as an unhealthy condition, which needs special attention, especially in the current outbreak of COVID-19. Based on the background, this study analyzed variables associated with the community psychosocial burden (anxiety level) during the COVID-19 pandemic in Indonesia.

## 2 Methods

### 2.1 Data source

The data collection was carried out by distributing an online survey to Indonesian citizens in 8 days (June 6th-13th, 2020). The final number of respondents was 8,031 respondents.

### 2.2 Variables

Psychosocial burden was determined based on anxiety level (Li et al., 2020; Moccia et al., 2020). The anxiety level was measured from 5 aspects, such as economic, religious, educational, employment, and social relationship aspects. Each question in the questionnaire used a Likert scale. The anxiety level was measured by adding up scores of those five aspects. The results were categorized into low, middle, and high category. This study has some limitations as it does not validate and measure the accuracy of data based on certain standards.

This study included 6 independent variables in the analysis, such as age group, gender, religion, marital status, education level, and employement. Age group was the respondent's last birth day categorized into ≤19, 20–29, 30–39, 40–49, and ≥50 years. While, gender was categorized into male and female. In terms of religion, the respondents were categorized into Moslem, Christian, Catholic, Hindu, and Buddist. Marital status were grouped into single, married, and divorced/widowed. Education level was the latest of education level, categorized into secondary and under, and higher education. Employement referred to the respondents' job. It was divided into unemployed, public servant/army/police, private sector, entrepreneur, farmer/fisherman/labor, and housewife.

### 2.3 Data analysis

All dependent and independent variables were dichotomous variables. In the initial stage, the researchers performed Chi-Square bivariate tests to examine the relationship of dependent and independent variables. In the final stage, a multivariate multinomial logistic regression test was performed to determine factors associated with anxietylevel. All statistical analyses were carried out using SPSS 22 software.

### 2.4 Ethical approval

This study has received an ethical approval from the National Ethics Commission (No: RK.05/KEPK/STIK/VIK/2020). While, the respondents' identities were removed from the dataset. The respondents provided a written approval for their involvement in the study.

## 3 Results

Table 1 shows the composite distribution of psychosocial burden variables. Education and employement trigger higher anxiety level in the respondents than other aspects do.

**Table 1:** The composite distribution of the community psychosocial burden during the COVID-19 pandemic in Indonesia, 2020 (n = 8,031).

No	Questions	1	2	3	4	5
1	How would you describe your feeling on your **economic** situation during the COVID-19 pandemic?	0.4%	2.6%	38.6%	44.1%	14.3%
2	How would you describe your feeling situation on your **religious life** during the COVID-19 pandemic?	1.6%	6.4%	37.1%	44.0%	11.0%
3	How would you describe your feeling on your or your children's **education** during the COVID-19 pandemic?	0.6%	2.4%	22.8%	52.0%	22.2%
4	How would you describe your feeling on your **work life** during the COVID-19 pandemic?	1.0%	3.0%	33.4%	47.0%	15.7%
5	How would you describe your feeling on social relationship during the COVID-19 pandemic?	0.5%	2.5%	30.7%	55.6%	10.6%

Note: 1 = not significantly worried; 2 = not worried; 3 = moderately worried; 4 = worried; 5 = very worried.

The co-linearity test of the psychosocial burden variables is presented in Table 2. The tolerance value of all variables was at > 0.10, and VIF value for all variables was at <10.00. These results show no symptoms of multicollinearity in the regression model.

**Table 2:** The results of the co-linearity test of the community psychosocial burden during the COVID-19 pandemic period in Indonesia, 2020 (n = 8,031).

Variables	Collinearity Statistics	
	Tolerance	VIF
Age group	0.469	2.134
Gender	0.954	1.048
Religion	0.994	1.006
Marital status	0.498	2.009
Education level	0.730	1.370
Employement	0.927	1.079

Dependent variable: The anxiety level.

Table 3 shows the statistical description of respondents’ characteristics. It can be seen that the respondents who experienced high anxiety level were dominated by those in the age group of 20–29 years and females.

**Table 3:** Statistical description of respondents’ characteristics (n = 8,031).

Variables	The Anxiety Level						P
	Low		Middle		High		
	n	%	n	%	n	%	
Age groups							∗∗∗< 0.001
• ≤ 19	2	1.7%	267	7.9%	414	9.1%	
• 20-29	25	20.7%	1148	34.1%	2044	45.0%	
• 30-39	26	21.5%	754	22.4%	1081	23.8%	
• 40-49	30	24.8%	674	20.0%	666	14.7%	
• ≥ 50	38	31.4%	527	15.6%	335	7.4%	
Gender							∗∗∗< 0.001
• Male	64	52.9%	1085	32.2%	1281	28.2%	
• Female	57	47.1%	2285	67.8%	3259	71.8%	
Religion							0.807
• Moslem	109	90.1%	2997	88.9%	4074	89.7%	
• Christian	5	4.1%	230	6.8%	272	6.0%	
• Chatolic	4	3.3%	80	2.4%	114	2.5%	
• Hindu	3	2.5%	58	1.7%	73	1.6%	
• Budhist	0	0.0%	5	0.1%	7	0.2%	
Marital status							∗∗∗< 0.001
• Single	24	19.8%	1328	39.4%	2126	46.8%	
• Married	91	75.2%	1952	57.9%	2292	50.5%	
• Divorced/Widowed	6	5.0%	90	2.7%	122	2.7%	
Education level							∗∗∗< 0.001
• Secondary and under	15	12.4%	1015	30.1%	2040	44.9%	
• Higher	106	87.6%	2355	69.9%	2500	55.1%	
Employement							∗∗∗< 0.001
• Unemployed	9	7.4%	558	16.6%	916	20.2%	
• Public servant/army/police	68	56.2%	1362	40.4%	1350	29.7%	
• Private sector/Professional	31	25.6%	927	27.5%	1426	31.4%	
• Entrepreneur	12	9.9%	297	8.8%	504	11.1%	
• Farmer/fisherman/labor	0	0.0%	27	0.8%	49	1.1%	
• Housewife	1	0.8%	199	5.9%	295	6.5%	

Note: ∗ P < 0.05; ∗∗ P < 0.01; ∗∗∗ P < 0.001.

Table 3 informs that the Moslem respondents dominated all anxiety levels. The respondents who have been married dominantly experienced all anxiety levels. From the perspective of education level, those with higher education level were dominantly at all anxiety levels.

Moreover, the respondents who worked in a private sector or professional job had high anxiety level. Low and middle anxiety level was dominated by those who worked as public servants, army, or police.

Table 4 displays the multinomial logistic regression of the psychosocial burden conducted using low anxiety level as a reference.

**Table 4:** The result of multinomial logistic regression of community psychosocial burden during COVID-19 pandemic in Indonesia, 2020 (n = 8,031).

Variables	The Anxiety Level							
	Medium				High			
	Sig	OR	Lower Bound	Upper Bound	Sig	OR	Lower Bound	Upper Bound
Age group: ≤ 19	0.282	2.578	0.460	14.453	0.067	5.006	0.895	28.005
Age group: 20-29	0.203	1.619	0.771	3.399	∗∗∗0.000	4.330	2.061	9.093
Age group: 30-39	0.053	1.679	0.992	2.841	∗∗∗0.000	3.802	2.240	6.453
Age group: 40-49	0.126	1.476	0.896	2.431	∗∗0.001	2.322	1.402	3.846
Age group: ≥ 50	-	-	-	-	-	-	-	-
Gender: Male	∗∗0.002	0.544	0.372	0.795	∗∗0.001	0.510	0.349	0.746
Gender: Female	-	-	-	-	-	-	-	-
Marital status: Single	0.115	2.402	0.808	7.139	0.505	1.447	0.488	4.295
Marital status: Married	0.285	1.612	0.671	3.872	0.526	1.328	0.553	3.189
Marital status: Widowed/Divorced	-	-	-	-	-	-	-	-
Education Level: Seconday	0.067	1.819	0.958	3.452	∗∗∗<0.001	3.117	1.646	5.904
Education Level: Higher	-	-	-	-		-	-	-
Employement: Unemployed	0.099	0.167	0.020	1.403	0.093	0.161	0.019	1.358
Employement: Public servant/army/police	0.055	0.142	0.019	1.043	∗0.037	0.120	0.016	0.884
Employement: Private sector	0.069	0.154	0.021	1.155	0.068	0.154	0.021	1.150
Employement: Entrepreneur	0.053	0.130	0.016	1.024	0.058	0.135	0.017	1.066
Employement: Farmer/fisherman/labor	∗∗∗<0.001	1392586.887	824529.062	2352007.136	.	1456079.053	1456079.053	1456079.053
Employement: Housewife	-	-	-	-	-	-	-	-

Note: ∗ P < 0.05; ∗∗ P < 0.01; ∗∗∗ P < 0.001.

Table 4 shows that male respondents were 0.544 times more likely to experience medium anxiety level than female respondents (OR 0.544; 95% CI 0.372–0.795). It means that males had a lower probability to experience the medium anxiety level than females.

Farmers, fishermen, or labors had the possibility of 1392586.887 times to be at the medium anxiety level compared to housewives (OR 1392586.887; 95% CI 824529.062–2352007.136). It indicates the respondents who worked as a farmer, fisherman, or labor had much higher chance of being at the medium anxiety level than a housewife.

With respect to age, the respondents aged between 20-29 years were 4,330 times more likely to experience high anxiety level than those aged ≥50 (OR 4,330; 95% CI 2,061-9,093). While, those in the 30–39 age group were 3,802 times more likely to bet at high anxiety level than those in the ≥50 age group (OR 3,802; 95% CI 2,240-6,453). The respondents who were 40–49 years had 2,322 times more possibilities of experiencing high anxiety than those in the ≥50 age group (OR 2,322; 95% CI 1,402-3,846). It explains older people tend to have lower possibility of experiencing high anxiety.

Table 4 also illustrates that male respondents were 0.510 times more likely to experience high anxiety than females (OR 0.510; 95% CI 0.349–0.746). It indicates males had a lower likelihood of high anxiety than females.

Additionally, the respondents with secondary and lower education levels had 3,117 times more possibilities of high anxiety than those with high education level (OR 3,117; 95% CI 1,646-5,904). In other words, those with higher education level were less likely at the high anxiety level.

The respondents who worked as public servants, army, police were 0.120 times more likely to feel highly anxious compared to housewives (OR 0.120; 95% CI 0.016–0.884). It means some employement status, such as public servant, army, or police had a lower likelihood of experiencing high anxiety.

## 4 Discussion

This study has some limitations as it does not validate and measure the accuracy of data based on certain standards. However, in general, the results of this study are in line with the previous research which found the severity of negative psychological impacts on psychiatric patients during the COVID-19 epidemic due to strict lockdown measures (Hao et al., 2020).

This study found that older ages tend to experience high anxiety level less frequently. In other words, younger age might overcome anxiety due to situations encountered during the pandemic. People with older age usually have experienced many events in their lives as they can learn from their past experience and adapt to new situations.

Other studies also show maturity and age were hypothesized to be positively related to subjective well-being, i.e, having the ability to overcome and adapt to existing problems and situations (Wang et al., 2020a, Wang et al., 2020b). Another article also found that older people generally could interpret events in their lives better than those younger (Wang et al., 2020a, Wang et al., 2020b).

Analyzed from gender, males had a lower probability to experience medium to high anxiety level than females. This study reveals that males could cope with stressful situations better during the pandemic than females. Identified from the aspects studied, only education and employement aspects could have more tendency to increase anxiety level.

These results reinforce another study, which found that males had more safe psychosocial condition (Kiely et al., 2019). The While, female respondents were more likely to experience mental disorders in the form of depression and anxiety than male respondents.

As previously said, it was no surprise that education became the determinant of anxiety level. Someone with a high education level could access the right information that calm his psychological condition (Hurwitz et al., 2008). A better education level can also make someone more careful and thoughtful about decision making from various perspectives (Tappe et al., 2010).

Some studies in several countries, such as in America (Banks et al., 2019), Lithuania (Kalediene and Petrauskiene, 2000), and in the Netherlands (Cloïn et al., 2011) informed the same findings. In general, some of the studies reported education level was directly proportional to indivdual's ability to respond to various situations in his life.

In another aspcet, employement is the other determinant of the community psychosocial burden during the COVID-19 pandemic. Farmers, fishermen, and factory workers who were involved in this study experienced quite high anxiety compared to other occupational groups. It may be due to job characteristics contrary to the existing health protocols applied, such as wearing masks, physical distancing, and avoiding crowds (Task Force for the Acceleration of Handling COVID-19 2020a). However, this study shows opposite finding to the previous research which found low anxiety in factory workers could be due to precautionary measures (Tan et al., 2020).

Those employments also cannot be restricted since the respondents who worked in those sectors had to meet their family needs (Baker, 2020).

While, other employments can be relatively modified and adjusted to the existing regulations (Kramer and Kramer, 2020). Among others include private employees who can work from home by changing some details of assigned tasks. Besides, public services also encounter some adjustments, for instance automation in its processes. In other words, people do not need to be present when they are in needs of the services.

The anxiety level can be reduced by using cognitive behavior therapy (Ho et al., 2020) to avoid more assigned serious health problems.

## 5 Conclusions

This study concluded that four variables affected the community psychosocial burden during the COVID-19 pandemic in Indonesia. These include age, gender, education, and employment. The results of this study are expected to encourage the Indonesian government to develop mental health programs during the COVID-19 pandemic.

## Declarations

### Author contribution statement

E. Nugraheni: Conceived and designed the experiments.

R. A. P. Geno: Performed the experiments.

A. D. Laksono, H. Megatsari: Analyzed and interpreted the data; Wrote the paper.

M. Ibad: Analyzed and interpreted the data.

K. P. Sarweni, Y. T. Herwanto: Contributed reagents, materials, analysis tools or data.

### Funding statement

This research did not receive any specific grant from funding agencies in the public, commercial, or not-for-profit sectors.

### Competing interest statement

The authors declare no conflict of interest.

### Additional information

No additional information is available for this paper.
